# Endothelial Extracellular Vesicles: From Keepers of Health to Messengers of Disease

**DOI:** 10.3390/ijms22094640

**Published:** 2021-04-28

**Authors:** Allison Mathiesen, Tyree Hamilton, Nigeste Carter, Michael Brown, William McPheat, Anca Dobrian

**Affiliations:** Department of Physiological Sciences, Eastern Virginia Medical School, Norfolk, VA 23501, USA; mathieah@evms.edu (A.M.); hamilttj@evms.edu (T.H.); carternm@evms.edu (N.C.); brownmt@evms.edu (M.B.); mcpheawl@evms.edu (W.M.)

**Keywords:** exosomes, microvesicles, EndMT, heterogeneity, miRNA, dysfunction, plasticity, cardiovascular disease, COVID-19

## Abstract

Endothelium has a rich vesicular network that allows the exchange of macromolecules between blood and parenchymal cells. This feature of endothelial cells, along with their polarized secretory machinery, makes them the second major contributor, after platelets, to the particulate secretome in circulation. Extracellular vesicles (EVs) produced by the endothelial cells mirror the remarkable molecular heterogeneity of their parent cells. Cargo molecules carried by EVs were shown to contribute to the physiological functions of endothelium and may support the plasticity and adaptation of endothelial cells in a paracrine manner. Endothelium-derived vesicles can also contribute to the pathogenesis of cardiovascular disease or can serve as prognostic or diagnostic biomarkers. Finally, endothelium-derived EVs can be used as therapeutic tools to target endothelium for drug delivery or target stromal cells via the endothelial cells. In this review we revisit the recent evidence on the heterogeneity and plasticity of endothelial cells and their EVs. We discuss the role of endothelial EVs in the maintenance of vascular homeostasis along with their contributions to endothelial adaptation and dysfunction. Finally, we evaluate the potential of endothelial EVs as disease biomarkers and their leverage as therapeutic tools.

## 1. Introduction

Endothelium is a key gateway for communication between blood and stroma. Recent evidence highlights the remarkable heterogeneity and plasticity of the endothelial cells in development, health and disease. The molecular signatures of the stromal cells in different organs are reflected within the transcriptome of their resident vascular endothelia. Endothelial functional and molecular zonation within various tissues shows how endothelial heterogeneity serves to optimize physiologic tissue function. In disease, endothelium can undergo dedifferentiation or transdifferentiation, thereby contributing to vascular maladaptation and dysfunction. The rich vesicular network of the endothelial cells designed to facilitate the regulated transport of hormones, growth factors, nutrients and pathogens also contributes to the biogenesis and uptake of extracellular vesicles (EVs). Due to its unique position as the interface between blood and lymph, the endothelium is a significant contributor to the circulating secretome. The particulate secretomes of endothelia mirror their molecular heterogeneity and may support plasticity and adaptation of endothelial cells within or between different vascular beds while also having significant functional impacts on circulating cells, including immune cells in blood or lymph. Therefore, EVs can support the physiological adaptation of endothelium or can contribute to the pathogenesis of vascular diseases and other diseases. In this review we will revisit the key evidence explaining endothelial plasticity and heterogeneity in health and disease. Herein, we discuss the role of endothelial EVs in the maintenance of vascular homeostasis, along with their contributions to endothelial plasticity and maladaptive remodeling, which are at the roots of the pathogenesis of cardiovascular diseases. Finally, we will explore the potential of endothelial EVs as disease biomarkers and their leverage as therapeutic tools.

## 2. Endothelial Plasticity and Heterogeneity

### 2.1. Roles in Development

Cellular identity is often thought of in terms of a stable state that is the terminal end product of differentiation. Cellular plasticity is the biological phenomenon whereby a terminally differentiated cell may take on a new specialized identity. This process can occur in response to physiological or pathological environmental cues and involves repression and activation of genes associated with the new cellular functions. Endothelial cells (ECs) are highly plastic, able to modulate their phenotype and function in response to stimuli in a variety of developmental, physiological and pathological processes. This plasticity in response to physiological cues and in disease results in a remarkable phenotypic heterogeneity. ECs arise from the mesoderm during embryonic development; they initially exhibit a non-specialized phenotype and then develop specialized identities specific to their vascular beds.

#### 2.1.1. Specification into Endothelial and Hematopoietic Lineages

**Figure 1:**
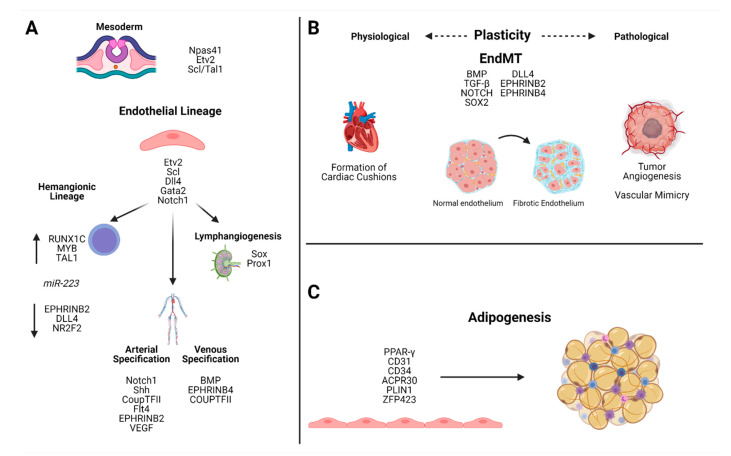
Specification into endothelial and hematopoietic lineage cells is driven through expression of Npas41 and Etv2. Further specification into hemangionic, arterial/venous and lymphatic cells is driven by distinct signaling pathways. Endothelial-to-hematopoietic transition is characterized by increased expression (↑) of RUNX1C, MYB, and TAL1 and reduced expression (↓) of EPHRINB2, DLL4, and NR2F2 and has been shown to require expression of miR-223 (**A**). EndMT is a process that is driven by transcriptional activation of epithelial or mesenchymal genes and is involved in both physiologic and pathologic endothelial remodeling (**B**). Adipogenesis is mediated in part by PPAR-gamma and results in reduced expression of endothelial markers and increased expression of adipocyte markers (**C**).

Endothelial cells and the vascular network emerge from a process known as vasculogenesis. Transcriptional control of vasculogenesis is regulated by a group of transcription factors including E26 transformation-specific (ETS). Specification into endothelial and hematopoietic lineages during embryogenesis is primarily driven through the expression of transcription factors Npas41 and Etv2. While it has been established in multiple studies that Etv2 is a major regulator of angioblast specification, recent studies have uncovered the regulatory role of the transcription factor Npas41. Expression of Npas41 has been shown to upregulate the endothelial and hematopoietic lineage markers (Etv2 and Scl/TaI1) in zebrafish [1] (Figure 1A). Etv2 is transiently expressed in early murine embryogenesis, and loss of expression in mice and zebrafish is catastrophic to vascular development. Etv2 has been shown to induce VEGF or Flk1 expression, and may function downstream of BMP, Notch and Wnt [2]. The exact pathways by which Etv2 mediates its vasculogenic activity are not yet fully understood, but recently it was shown using chromatin immunoprecipitation-sequencing analysis that there are multiple potential downstream target genes involved, including Scl, Gata2, DII4, Notch1 and Flt4 [3]. (Figure 1A). The embryonic vasculature is further differentiated into arterial, venous, lymphatic and hemogenic subtypes through activation of distinct signaling networks that remain under investigation. Arterial-venous specification is driven by a number of signaling pathways and transcriptional regulators. Both SoxF and VEGF signaling pathways have been implicated in the acquisition of arterial identities through targeting of the downstream targets Notch, BMP and TGF-beta. Notch1 is a downstream target of both VEGF and Sox. Loss of Shh, the upstream regulator of VEGF, results in impaired arterial differentiation [4]. Overexpression of Sox has been shown to lead to upregulation of both Notch signaling components and arterial markers and downregulation of venous markers [5]. Arteriovenous specification can be observed following amputation of the adult zebrafish caudal fin; the initial vascular plexus is principally venously derived; however, Notch-mediated conversion of venous-to-arterial cell fate permits comprehensive regeneration of the fin vasculature [6] (Figure 1A). Venous specification is less well understood. Venous-specific downstream signaling is driven by BMP and chicken ovalbumin upstream promoter-transcription factor II (COUP-TFII) [7]. BMP signaling mediates the specification of venous identity through SMAD1/SMAD5 interaction in both mice and in zebrafish [8]. Coup-TFII is expressed in venous, but not arterial endothelium and has been shown to suppress Notch signaling. Ablation of venous COUP-TFII results in the acquisition of arterial markers in transgenic mouse embryos [9] (Figure 1A).

Hematopoietic stem and progenitor cells (HSPCs) arise from a subset of endothelial cells, the hemogenic ECs (HemECs) that are specified during embryogenesis in a process called endothelium-to-hematopoietic transition (EHT). EHT is a Notch-dependent process that is distinct from arterio-venous specification, despite its early, close association with embryonic arterial vasculature. Human embryonic stem cells (hESCs) sorted by their tetraspanin signature, have revealed a population of hemogenic endothelial cells characterized by a lack of CD184 and CD73 concomitant with high expressions of hematopoietic genes, including RUNX1C, MYB and TAL1, and reduced expression of arterial endothelial genes, such as EPHRINB2 and DLL4 and of NR2F2—the master regulator in venous endothelium [10]. The determinants governing the specification of HemECs and the subsequent initiation of EHT are under current investigation. A recent publication that interrogated HemEC determination and progression through EHT in a zebrafish model, revealed that miR-223 mediated N-glycosylation signature of EC is required for HemEC progression through EMT [11] (Figure 1A).

The lymphatic vasculature is distinct from the vasculature of the circulatory system and is required for maintenance of fluid homeostasis, immune cell trafficking and absorption of lipids. The mechanisms of lymphatic endothelial cell specification are a matter of continuing investigation; however, several transcriptional regulators have been identified, including Sox and Prox1. While several members of the SoxF family are implicated in arterio-venous specification, some studies have suggested that they are also key players in lymphatic specification [2]. Sox18 acts as a molecular switch that activates Prox1, which is essential to both the differentiation and the maintenance of a lymphatic specification (Figure 1A).

#### 2.1.2. Organotypic Specification of Endothelium as a Source of EC Heterogeneity

Further EC specialization occurs in a tissue-specific manner whereby both EC and external tissues engage in directing phenotypic adaptations necessary for organ development and function [12,13]. This molecular and functional heterogeneity results in highly specialized ECs with distinct functions. For example, endothelial layers of the kidney glomeruli or intestinal mucosa are fenestrated to permit filtration or secretion, while the endothelium of the blood–brain barrier is continuous and specialized to restrict transcellular movement of solutes [14]. This specialization is reflected in molecular variations of EC from different tissue beds. Proteomic analysis of vascular beds from the aorta, liver and brain in C57Bl6 mice revealed distinct proteomic signatures differentiating the vascular ECs [15]. Then distinct populations of cardiac endothelial cells have been identified in the heart alone, as revealed by single cell RNA-seq characterization of adult human heart cells [16]. These EC populations included capillary, capillary-like, arterial, venous, atrial, lymphatic, cardiomyocyte-like and fibroblast-like EC populations [16]. In order to further characterize EC heterogeneity across distinct vascular beds, Jambusaria et al. employed a transgenic mouse model expressing an EC-specific ribosomal HA tag [17]. Transcriptional profiling using RNA-Seq followed by principal component analysis revealed distinct separation between clusters in an organ-specific manner, and differential expression analysis revealed an upregulation of genes previously thought to be uniquely translated in the parenchyma, but not in the endothelium [17].

Endothelial cells can revert to a mesenchymal phenotype, a process in which the endothelial cells lose their tight junctions, develop increased motility and increase production of extracellular matrix proteins in a process termed endothelium-to-mesenchymal transition (EndMT) [18]. EndMT is critical for heart valve development; endothelial cells undergo EndMT and migrate into the cardiac jelly, thereby forming endocardial cushions that will lead to formation of the valves and septa of the mature heart [19]. EndMT is driven by multiple signaling pathways, including BMP, TGF-β and Notch (Figure 1B) [20].

A number of microenvironmental cues, including laminar shear stress and cyclic strain, can induce remodeling of the vascular endothelium. Patterns of flow differ throughout the vascular tree, ranging from a low, constant flow in the veins, to high, pulsatile flow in arteries; laminar flow has a stabilizing effect on ECs, and both blood and lymphatic ECs align themselves in the direction of flow. Under situations of high shear stress however, the ECs will align perpendicularly to the flow. This axial polarization is mediated in part by vessel wall shear stress and varies by endothelial subtype [21,22]. Endothelial gene expression is also mediated by flow, resulting in region-specific phenotypic differences; and changes in flow pattern induce molecular and morphological differences among ECs. 

Revascularization following injury, particularly cardiac injury, is imperative for maintaining normal organ function. Delay or impairment of newly formed vessels can result in the formation of fibrotic scars. Unlike embryonic vascularization however, the formation of new vasculature following an injury is due to the sprouting of nearby vessels rather than the transdifferentiation of neighboring endothelial cells [23,24,25].

### 2.2. Roles in Pathophysiology: De-Differentiation, Proliferation and Transdifferentiation

Pathological endothelial plasticity has been implicated in multiple disease processes (Figure 1B). EndMT leads to a progressive loss of endothelial identity that is associated with various vasculopathies. EndMT has been observed in the endothelial cells of the blood–brain barrier, resulting in cerebral arteriovenous malformations caused by excessive Sox2 signaling [26]. In atherosclerosis, neointimal hyperplasia is an early feature of fibro-proliferative vascular disease. Neointimal lesions exhibit shear stress-induced EndMT [27]. EndMT also contributes to maladaptive remodeling in the microvascular endothelium of obese adipose tissue (AT) in humans, leading to a loss of barrier function and angiogenic capacity [28]. In proliferative diabetic retinopathy, pathologic differentiation within the lymphatic endothelium may drive aberrant neovascularization and ischemia [29]. Pathologic lymphangiogenesis has also been implicated in organ rejection, inflammation and tumorigenesis [30]. Blockage of lymphangiogenesis in cardiac allograft patients reduced rejection rates, potentially by reducing lymphatic facilitation of antigen presentation [31]. Lymphatic endothelial remodeling has also been implicated in a variety of cancers, and lymphatic infiltration of tumors facilitates tumor cell entry into the circulation and increases the incidence of lymphatic metastasis [30,32,33].

Formation of tumor vasculature is required for tumor growth and is achieved via different modes of vascularization, including endothelial sprouting, post-natal vasculogenesis by endothelial precursor cells (EPCs), intussusception (vessel splitting), co-option of existing vasculature, tumor cell differentiation into “endothelium-like” cells and vascular mimicry (VM) [34]. Vascular mimicry was first described in intraocular and metastatic melanoma cells; it is a process of dedifferentiation of tumor cells into an endothelium-like phenotype, enabling the formation of vascular-like channels [35]. VM provides a mechanism for tumor nourishment, as it allows vascularization even in the presence of anti-angiogenic therapies. The molecular mechanisms responsible for VM are not yet elucidated and a greater understanding may prove invaluable to future cancer treatment. Tumor angiogenesis is driven primarily by hypoxia; in hypoxic conditions, cancer cells secrete VEGFA, which engages the VEGFR2 receptor on nearby vascular endothelial cells, stimulating the growth of new vascular sprouts [36] (Figure 1B).

Postnatal transdifferentiation of EC into functional terminally differentiated cells is a matter of continued debate. The most prominent example is provided by studies that examined transdifferentiation of endothelial cells into adipocytes. Studies of both murine and human adipose tissue show that a subset of adipocyte progenitor cells reside in the adipose tissue vasculature. While the phenotype of these cells remains contested, there is evidence to support the identities of these precursors as endothelial cells, smooth muscle cells/pericytes or some gradation of intermediate phenotypes [37,38]. Lineage tracing of the EC promoter VE-cadherin in brown and white adipose tissue of embryonic and post-natal mice revealed that the EC promoter VE-cadherin was expressed in brown and white adipocytes at some point in their development [39]. Rosiglitazone, a PPAR-γ agonist, has been proven to be an effective inducer of adipogenesis [40,41]. Exposure of murine thoracic aortic rings to rosiglitazone resulted in increased accumulation of lipid droplets, reduced expression of endothelial genes (CD31 and CD34) and an increase in adipocyte-specific genes (Acrp30 and Plin1); and the preadipocyte marker Zfp423 has been found in both mural and vascular endothelial precursors [39,42,43,44]. The concept of endothelial cells as adipocyte precursor cells is not without controversy, however. Some studies have been unable to demonstrate the presence of endothelium-cell derived adipocytes in mice fed either normal of high fat diets [45] (Figure 1C).

## 3. Extracellular Vesicles and Their Roles in Endothelial Plasticity and Heterogeneity

### 3.1. Basic Concepts of Extracellular Vesicles

**Figure 2:**
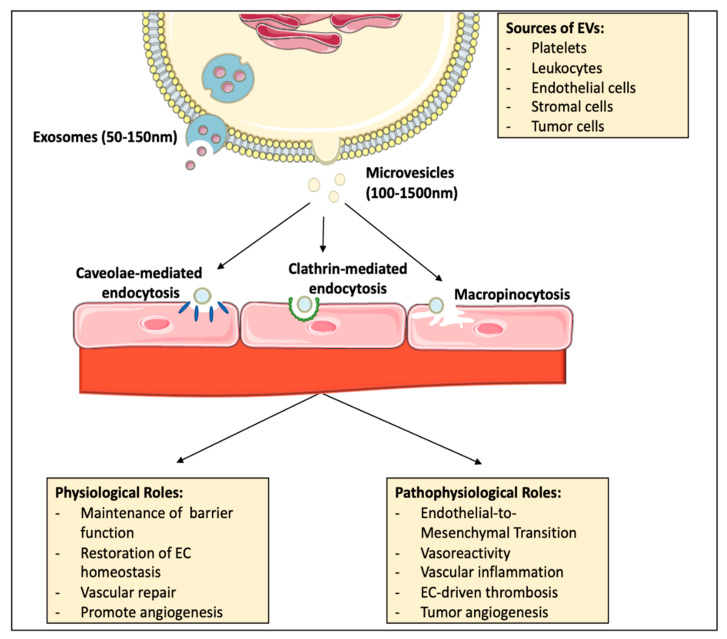
Exosomes and/or microvesicles can be released into the extracellular space by virtually all cell types. Once in circulation, EVs can be taken up by the endothelium via macropinocytosis, clathrin-mediated or caveolae-mediated endocytosis. EVs can deliver cargo that contributes to the plasticity of recipient endothelial cells, resulting in physiological or pathophysiological changes.

Cell–cell communication plays an important role in the overall maintenance of homeostatic conditions in various tissues. One particular type of cell communication is via secretion of EV, a heterogenous population of lipid bilayer encapsulated cellular cargo. EVs are released by virtually all cell types under both basal and pathological conditions, and contain unique collections of lipids, proteins, microRNAs and other noncoding RNAs and metabolites which can contribute to the modulation of biological function in recipient cells. This cargo can be heavily influenced by the metabolic state of the parent cell, stimuli in the microenvironment and the biogenesis of the vesicle. EVs can be categorized as exosomes or microvesicles, based on their mechanisms of biogenesis (Figure 2). Exosomes are generated due to the inward budding of multivesicular endosomes in the endosomal compartment, thereby forming intraluminal vesicles. Mature multivesicular endosomes will then translocate to the plasma membrane, where they will fuse with the lipid bilayer, releasing the intraluminal vesicles as exosomes. Exosomes (also denominated as small EVs) typically have diameters ranging between 50 and 150 nm [46]. Exosome biogenesis largely depends on the mechanisms necessary to facilitate the sorting and packaging of targeted cargo. Specifically, cargo selectively incorporated into intraluminal vesicles and targeted for secretion via exosomes, is generally recycled from the plasma membrane or delivered from the trans Golgi network [46]. During exosome biogenesis, cargo sorting can be mediated by several factors, including post-translational modifications of target proteins, such as ubiquitylation [47,48]. These modified proteins can then be recognized by the endosomal sorting complexes required for transport (ESCRTs), in which the ESCRT0 and ESCRTI complexes cluster the ubiquitylated cargo on microdomains of the limiting membrane of multivesicular endosomes [46,49]. This clustering leads to the activation of ESCRTII, which then recruits ESCRTIII complexes necessary for vesicle budding and fission. Furthermore, vesicle cargo can be recruited in a ubiquitin-independent manner via the accessory protein ALIX, a protein widely used as an exosomal marker due to its abundance in small vesicles [50]. There are also several ESCRT-independent mechanisms that can facilitate cargo sorting, such as the conversion of sphingomyelin into ceramide by neutral type II sphingomyelinase. Ceramide can then be further metabolized into sphingosine-1-phosphate which binds to inhibitory G protein (Gi)-coupled S1P receptors found on multivesicular endosomes. This signaling event has been shown to be critical for cargo sorting into exosomal intraluminal vesicles [51]. Microvesicles are generated due to the outward budding of the plasma membrane. This mechanism is characterized by cytoskeleton remodeling and the rearrangement of lipid composition of the plasma membrane due to increased activity of the enzymes flippase, floppase and scramblase [52]. Microvesicle diameter can range between 100 and 1500 nm [53]. Unlike exosomes, the exact mechanisms for cargo sorting into microvesicles are less understood. Despite this, is has been shown that components of the ESCRT machinery, such as the ESCRT-associated ATPase Vps4 and TSG101, may also be involved in protein recruitment and sorting into microvesicles [54] Furthermore, studies have found that cargo can also be targeted to the site of microvesicle formation due to their affinity for lipid rafts or by direct anchoring to plasma membrane lipids [55,56,57]. Given the overlapping properties of exosomes and microvesicles, for the remainder of the review we will collectively refer to them as EVs, nomenclature recommended by the International Society of Extracellular Vesicles (ISEV) in 2014 [58].

### 3.2. EV Circulation and Uptake by the Endothelium

The cardiovascular and lymphatic systems converge to make up an extensive circulatory network of vessels and organs. The major role of the cardiovascular system is to deliver nutrients and oxygen to tissues and organs while also removing waste [59]. The lymphatic system supports the circulatory system by draining excess interstitial fluid from tissues back into the blood. In both systems’ vessels, the lumen is composed of a thin, single layer of endothelial cells that forms a barrier between circulating blood or lymph in the lumen and the vessel wall. Therefore, by being ubiquitously located throughout this extensive circulatory network, endothelium is continuously exposed to macromolecules and EVs that circulate between the two compartments. The lymphatic and blood vessel networks have multiple points of convergence, allowing EVs from various tissues to travel into the bloodstream. In fact, EVs can be detected in various biological fluids, including but not limited to blood, urine, saliva, cerebrospinal fluid and breast milk [60]. EVs in these biological fluids can then enter systemic circulation and elicit different responses in various cell types in distant tissues. It can be postulated that, together with immune cells and platelets in circulation, the endothelium is the type of cell most exposed to EVs and is the gatekeeper for the movement of EVs between the fluids and tissue compartments. Interactions of EVs with endothelium are the subjects of intense research, as they have important implications for understanding the mechanisms that control the selectivity and permittivity of such interactions. With the use of high-speed imaging, EVs have been shown to roll, arrest and accumulate on the endothelial cell walls of veins in vivo [61,62]. Furthermore, the endothelium contains junctional zones together with apical and basolateral domains, providing not only a barrier function, but also a means of transport for signaling molecules by the well-characterized mechanisms of endothelial cell transcytosis [63,64]. It has been previously shown that both tumor and adipocyte-derived EVs can be transported via this mechanism across endothelial cells of the blood–brain barrier or the circulatory system, respectively [63,65]. Once EV come in contact with the endothelium, they can be taken up through several classical pathways, including macropinocytosis, clathrin-mediated endocytosis and caveolae-mediated endocytosis (Figure 2). Using imaging flow cytometry, Banzis et al. demonstrated that EV uptake by mouse aortic endothelial cells significantly decreased when treated with pharmacological inhibitors targeting clathrin-dependent endocytosis, caveolin-1 related uptake and acidification of the endocytic vesicles, consequently inhibiting micropinocytosis [66]. Furthermore, the internalization of EVs via endocytosis has also been shown to be mediated by interactions between proteins embedded within EV membranes, and endothelial cell membrane receptors. Specifically, Rana et al. confirmed that Tspan8-α4 complex-bearing EVs were internalized by rat aortic endothelial cells by binding to intercellular adhesion molecule (ICAM)-1 [67]. Moreover, blocking membrane proteins such as α4 integrin and CD29 can inhibit the internalization of microvesicles by human microvascular endothelial cells [68]. This suggests that EV internalization may be a selective process. Moreover, these membrane proteins may also serve as molecular barcodes, targeting EVs towards specific recipient cells, and thus playing a role in the biodistribution of EVs once they enter the circulation. Using lipophilic labeled EVs, Whitham et al. found that EVs released into circulation during exercise localized to the liver [69]. The authors attributed differences in the localization of EVs isolated during exercise and at rest to adhesion proteins within the vesicle membrane, such as integrins and tetraspanins, which may target specific organs. Additionally, EV biodistribution in vivo, in mice, is dependent on the cellular source with preferential accumulation in the liver, the spleen, the gastrointestinal tract or the lungs [70]. Regardless of their final destination, upon internalization, EVs can either fuse with an early endosome or fuse with the plasma membrane, thereby releasing their contents into the intraluminal vesicles, the cytosol or the nucleus, leading to functional changes within the cells of the recipient tissues.

### 3.3. EVs as Contributors to Endothelial Physiology

Currently, there is little known about the physiological roles of EVs; however, some literature suggests that EVs may play a role in the homeostatic maintenance of the endothelium. The protection and repair of the endothelium are essential processes for the preservation of EC integrity and functions [71]. While little was previously known about the mechanisms by which the endothelium is repaired and regenerated, investigators demonstrated that injured ECs can be protected from complement-induced apoptosis by shedding EVs containing caspase-3 [72]. Thus, the release of EVs containing caspase-3 protects the endothelium against stress. In the context of chronic endothelial dysfunction, investigators found that EC-EVs are incorporated into recipient endothelium in an annexin/phosphatidylserine-dependent manner and protect the recipient endothelium against apoptosis by inhibiting p38 activity [73]. Furthermore, it has been shown that endothelial cell-derived EVs are released in response to extracellular stimuli that trigger changes in phenotype and tissue remodeling. In vitro, microvascular ECs cultured in hypoxic or inflammatory stress conditions release EVs containing proteins and mRNA that reflect the state of the parental cell. Specifically, EVs derived from TNFα treated EC contained cargo associated with anti-oxidant protection and immune response [74]. This suggests endothelial cells under stress may communicate these conditions to neighboring cells by means of EVs. It has also been shown that EC-derived EVs can work in an autocrine manner to restore EC homeostasis. Mahmoud et al. found that EC-derived EVs delivered endothelial nitric oxide synthase (eNOS) following fatty acid-induced oxidative stress [75]. Additionally, EVs have been shown to be instrumental for endothelial repair and plasticity in many contexts, including wound healing, neuronal recovery following traumatic brain injury, cell cycle regulation and tumorigenesis. EV derived from adipose-derived stem cells have been shown to promote angiogenesis; to accelerate wound healing of full-thickness skin wounds in mice; and to promote the survival of fat grafts by stimulating angiogenesis, proliferation and migration in nude mouse models [76,77]. Neuronal stem cell-derived EVs have been demonstrated to facilitate functional recovery of spinal cord injuries by stimulating angiogenesis in mouse models [78]. In rat models of traumatic brain injury, mesenchymal stromal stem cell EV treatment improved spatial learning and sensorimotor function and led to a significant increase in the generation of new endothelial cells [79].

According to Hunter et al. platelet-derived EVs are the most abundant type of vesicle found in circulation under basal conditions, and thus may also contribute to maintenance of the endothelium. Platelet-derived EVs have been shown to play a role in stabilizing the vasculature and maintaining endothelial cell barrier function [80]. Miyazawa et al. demonstrated that pulmonary endothelial cells displayed increased barrier function and maintenance of junctional proteins such as VE-cadherin and ZO-1 when pretreated with platelet-derived EVs following stimulation with thrombin, a known inducer of vascular permeability [81]. Furthermore, it has been demonstrated that platelet-derived vesicles can induce vascular endothelial growth factor (VEGF)-dependent angiogenesis and stimulate post-ischemic revascularization following chronic ischemia [82].

It has also been shown that EVs play a role in fetal development. During pregnancy, there is an increase in metabolic demands for the mother and fetus; thus, physiological adaptations in the cardiovascular system are necessary to ensure adequate nutrient and oxygen supplies. Hoegh et al. reported that placental microvesicles may induce changes in the transcriptomes of endothelial cells, impacting genes involved in endothelial proliferation [83]. In vitro analysis demonstrated that exosomes from pregnant women could also stimulate endothelial cell migration [84]. Despite the ongoing research on the physiological role of EVs, their role in normal development and adult tissue homeostasis are still far from being comprehensively understood.

### 3.4. Contribution of EVs to Pathogenic Endothelial Plasticity and Dysfunction

#### 3.4.1. The Role of EVs in EndMT

EVs have been implicated in the induction of EndMT in multiple pathological conditions. Human umbilical vein endothelial cells (HUVECs) undergo EndMT when exposed to melanoma-derived EVs [85]. Urinary EV obtained from rat models of chronic kidney disease were found to be enriched with miRNA implicated in inflammatory processes and EndMT [86]. There are multiple reports of EV-induced EndMT associated with obesity and metabolic syndrome. Adipose tissue in obese conditions is a rich source of EVs that can act as messengers between cells of the proinflammatory obese environment and endothelial cells of distant vascular beds. EVs obtained from adipose tissue of patients undergoing bariatric surgery were found to contain protein and miRNA cargo that reflect the proinflammatory signatures of their parent cells. These EVs have been shown to induce EndMT in adipose microvascular endothelial cells in vitro [28]. EVs derived from murine adipocyte cells exposed to the proinflammatory cytokine TNFα, induced an increase in markers consistent with EndMT in HUVECs along with increased leukocyte attachment [87].

#### 3.4.2. EVs and Modulation of Angiogenesis

Pathologic angiogenesis, a hallmark of various proliferative diseases, was shown to be impacted by EVs particularly via their miRNA cargo. Tumor angiogenesis, a critical step in malignant cell survival and proliferation, was shown to be supported by EVs released by tumor cells. Several studies have shown that tumor cell-derived EVs contain VEGF, promoting EC migration and angiogenesis in vitro [88,89,90,91]. Giusti et al. found that vesicles secreted by glioblastoma cells contained VEGF and thus led to increased wound closure and tube formation when applied to human brain microvascular EC [90]. Similarly, glioblastoma stem-like cell EVs transport VEGF-A, contributing to more robust angiogenesis in human brain endothelial cells in vitro [91]. However, the delivery of VEGF to recipient EC via EVs is not unique to tumor-derived EVs. Other studies have shown that plasma EVs from patients with diabetes mellitus contained increased VEGF-A levels compared to euglycemic individuals and can therefore contribute to excessive retinal angiogenesis in diabetic patients with retinopathies [92]. Furthermore, the transfer of miRNA via EVs has also been shown to promote angiogenesis. Matsuura et al. found that hepatocellular carcinoma cells, in hypoxic conditions, release EV containing miR-155 that stimulate HUVEC tube formation [93]. Mazzeo et al. found that plasma EVs from patients with diabetic retinopathy induce features of retinopathy within the retinal EC in vitro, including increased EC migration, permeability and tube formation [94]. Specifically, the authors identified several miRNAs that were up-regulated in EVs, some of which are known to be involved in angiogenesis and inflammation (miR-21-3p), and in cell migration (miR-21-3p and miR-17-5p). Additionally, the authors showed down-regulation of miR-150-5p and miR-342-3p, known to be anti-angiogenic. Tumor-cell-derived EVs can also have angiostatic effects. EVs released from two different oral squamous carcinoma cell lines had opposing pro and anti-angiogenic effects on HUVECs in vitro that were attributed to differences in the response to hypoxia of the parent cell [95]. Circulating EVs in the blood of patients diagnosed with multiple myeloma (MM) were found to be heavily enriched with the PIWI-interacting RNA, piRNA-823. These EVs transferred piRNA-823 to EA.hy926 EC treated with MM-derived EVs; and increased the expression of both VEGF and ICAM1, and promoted invasion, proliferation and tube formation in vitro, similarly to EA.hy926 transfected with a piRNA-823 mimetic [96].

#### 3.4.3. The Role of EVs in Endothelial Inflammation

Inflammatory mediators such as TNFα, interleukin (IL)-1β and thrombin enhance the generation of EC-EVs, which can in turn disseminate vascular inflammation in a paracrine fashion, hence reinforcing the cyclical inflammatory milieu [97]. One study found direct correlations among EC-EV numbers and soluble IL-6 and C reactive protein concentrations in healthy young men, indicating that low-grade inflammation and EC-EV formation are positively associated [98]. Low grade chronic inflammation is also a hallmark of obesity and is likely causal for comorbidities including cardiovascular disease [99]. EVs derived from inflamed endothelium of clinically obese individuals have been shown to increase VCAM-1 production in HUVECS and to enhance leukocyte attachment in vitro, which supports endothelial dysfunction [87]. Another in vivo study demonstrated that EC-EVs and naïve endothelial cells triggered by proinflammatory stimuli can be characterized by the upregulation of ICAM-1 mRNA expression and soluble ICAM-1 shedding from recipient cells [100]. Moreover, EC-EV biogenesis triggered by treatment with TNFα in vitro led to increased production of soluble ICAM-1 from recipient endothelial cells, thereby providing a positive forward loop that enhanced endothelial response to inflammation [101]. Proinflammatory cytokines can promote endothelial damage and barrier dysfunction, and EC-EVs that encapsulate proinflammatory cytokines elicit immune cell migration to sites of acute and chronic vascular endothelial damage [102]. Another study showed that EC-EVs collected from inflamed endothelial cells, once injected intravenously in mice, lead to systemic and pulmonary increases in IL-1β and TNFα, and increased neutrophil recruitment to the lungs [103]. Collectively, these studies suggest that EVs can effectively perpetuate inflammatory signals in a paracrine fashion, eliciting dysfunction in ECs that are not locally exposed to inflammation.

#### 3.4.4. Contributions of EVs to EC-Driven Thrombosis

Evidence in support of the thrombogenic role of EC-derived EVs is rather sparse. One paper by Leroyer et al. showed the involvement of EC-EVs in thrombin generation [104]. More recently, EC-EV release in circulation appears to play a significant role in the development of thrombi in combination with the activation of coagulation pathways [105]. Phosphatidylserine expressed externally on the surface of EC-EVs facilitates binding to recipient endothelial cells and activation of coagulation factors, thereby providing a potential mechanism for procoagulant effects of EC-EVs [106]. In particular, tissue factor (TF) is the major cellular activator for the extrinsic clotting cascade, and several in vitro studies found that different agonists induce TF-containing EVs’ pro-coagulant activity [107]. Immunological assays have identified TF within the EC-EVs’ cargo, which can lead to activation of the extrinsic coagulation pathway by recipient endothelial cells. This finding suggests that EC-EVs loaded with TF can initiate the assembly of clotting factors, leading to thrombin generation [108]. TF was previously thought to be expressed on the endothelial surface when vascular integrity was compromised. Recent evidence shows that, to some extent, TF is also transported inter-cellularly via EVs [109]. The ability of EC-EVs to mediate thrombin generation was first demonstrated in vitro by reduction of clotting time in normal plasma incubated with increasing amounts of EC-EV containing TF [110]. The thrombogenic activity of EC-EVs was also shown to be TF-dependent in murine models both by thrombin formation in vitro and thrombus formation in vivo [111,112]. TF-positive EC-EVs expressing endothelial adhesion molecules also can bind to other cell types, such as circulating monocytes, and effectively transfer bioactive TF in vitro [111]. EC-EVs can also serve as docking stations for assembly of the clotting cascade components, via interaction with positively charged γ-carboxyglutamic acid (GLA) domains on some clotting proteins [113]. Procoagulant EC-EVs positive for TF have also been found in atherosclerotic plaques [114]. Treatment of TF containing EC-EVs with an antibody against TF drastically reduced EV thrombogenicity [115].Contemporary research has identified a threshold of EC-EVs containing TF to negatively impact pulmonary lung capacity and exacerbate the severity of disease for individuals infected with COVID-19 [116]. Furthermore, EVs derived from TNF-stimulated HUVECs induced coagulation in vitro, via a tissue factor/factor VII-dependent pathway and increased the expression of E-selectin, ICAM-1, avβ3, and PECAM-1 showing that EVs can simultaneously increase coagulation and cell adhesion [108]. Finally, EC-EVs can enhance in situ thrombogenicity via their membrane proteases, such as TACE/ADAM17, which can cleave transmembrane proteins on the target endothelial cell surfaces, inducing the shedding of endothelial protein receptor EPCR [117].

#### 3.4.5. The Role of EVs in EC-Driven Vasoreactivity

In pulmonary vasculature, endothelial cells contribute to regulation of vessel diameter that allows control of pulmonary arterial blood pressure and total vascular resistance [118]. It is well recognized that the regulation of vasoreactivity by the endothelium is predominantly accomplished by paracrine signaling with smooth muscle cells through the release of various vasoactive agents such as nitric oxide and endothelin-1 [119]. Paracrine signaling not only involves the cross talk of soluble molecules through myoendothelial gap junctions, but also via EVs [120]. Under pathological conditions, the interaction between ECs and EVs may be chronically altered so that a sustained increased in vaso-contractility and abnormal vascular proliferation develops, which leads to high pulmonary artery pressure, vascular remodeling, ventricular hypertrophy and pulmonary hypertension [121]. Furthermore, vasoconstrictor-induced reactive oxygen species produced in vascular smooth muscle cells may diffuse into endothelial cells via EVs to reduce nitric oxide bioavailability, thereby diminishing endothelium-dependent control of vasoreactivity [122]. The dysfunctional endothelium displays to varying degrees, an imbalance in production of vasoactive mediators, leading towards excesses of vasoconstriction and pulmonary vascular remodeling [123]. Increased concentrations of different subsets of circulating EC-EVs were reported in pulmonary hypertension compared to control subjects; the profiles mirrored increased cellular activation and/or apoptosis [124]. The levels of VCAM and PECAM in EC-EVs were also correlated with hemodynamic parameters that reflect pulmonary vascular remodeling [124]. Moreover, in vivo studies have suggested that circulating EC-EVs in rats with hypoxia-induced pulmonary hypertension can modulate overall vascular tone and endothelial barrier integrity [125]. These findings revealed that EC-EVs compromise the endothelium-dependent vasorelaxation in pulmonary arteries ex vivo and decrease the nitric oxide production by pulmonary endothelial cells via increased oxidative stress.

## 4. Experimental Approaches for the Study of EV and EV-Cell Interactions

### 4.1. EV Isolation, Uptake and Intracellular Fate

As discussed in the previous chapter, EVs cannot not be regarded anymore as innocent bystanders in our efforts to understand various physiologic and pathophysiologic mechanisms. The increasing number of studies highlighting the biogenesis, targeting and interaction of EVs with cells in virtually every tissue prompted efforts to develop adequate tools to allow a more granular interrogation of EV biology. Two major challenges are EV heterogeneity and their small size. The latter makes visualization challenging using conventional light microscopy techniques, such as confocal microscopy, due to limits in resolution. The ability to accurately visualize and track single EVs will help mitigate the current challenges of attributing measurable effects to sub-populations of EVs that are more homogeneous in size, cargo or surface molecules.

Other important considerations in the EV field are the approaches used for their isolation and quantification, along with the amounts needed to generate information related to their cargo, uptake or intracellular delivery and molecular targets. A variety of methods that include ultracentrifugation, density gradient centrifugation and size exclusion chromatography are commonly used to isolate EVs [126]. These methods are time consuming, labor intensive, can impact EV yield and can contaminate with lipoproteins. Concerning evidence has demonstrated that isolation methods that result in contamination of the EV fractions with particulate or soluble factors can cause the misattribution of observed cellular effects to EVs [127].

As mentioned before, methods to visualize EVs are also challenging. Being able to visualize EV internalization by EC is difficult, especially for the small EVs in the <100 nm size range. Confocal microscopy is the most commonly used approach to study EV–cell interactions, such as internalization and cellular fate. However, very few of the current studies report individual EV internalization, but rather the presence of labelled EVs in intraluminal vesicles, which are much easier to visualize due to their larger average size. Examples of studies using such approaches include analyzing the differences in internalization of EVs (produced by cardiomyocytes), by human cardiac microvascular endothelial cells, human iPSC-derived cardiomyocytes and human cardiac fibroblasts [128]; and in the uptake of glioma derived EVs by HUVEC cells by co-staining EVs with calnexin and PKH67 [129].

Advances in super resolution microscopy (SRM) shifted the diffraction limit of fluorescent microscopy and allow for increased resolution down to 20 nm [130]. SRM can be used to visualize individual EV internalization in live cells [131], and circumvents the need to introduce artifacts related to the need to fix the cells as required by conventional electron microscopy. It has not yet been widely adapted due to the limited availability of fluorophores with robust brightness and stability, but future studies of endothelial cell EV interaction can greatly benefit from the utilization of SRM imaging.

Imaging flow cytometry offers the unique advantage of combining fluorescent imaging with the high throughput capabilities of conventional flow cytometry, which can address the heterogeneity of EV–cell interactions and the potentially different routes of uptake. The accompanying analysis software IDEAS offers guided wizards and a unique mask feature that allows for quick quantification of EV internalization and spot counts to determine the number of EVs inside the cell. Imaging flow and IDEAS software ( Amnis Corporation, Seattle, WA, USA) have been used to show the internalization of EVs derived from myeloma cells by endothelial cells and their subsequent impact on angiogenesis [132]. Imaging flow cytometry has also been utilized to monitor tagged protein and RNA cargo uptake in EVs derived from cells infected with *Plasmodium falciparum* [133].

EV labelling is required for all the above applications, and increasing concerns are related to the validity of many of the commonly used dyes. Lipophilic dyes such as DiD and PKH have been criticized for the potential of false positives due to dye aggregates and the labeling of lipoproteins that can co-isolate with EVs during certain isolation procedures [134]. Fluorescently-tagged cargos can be transfected into EVs, and while this technique offers greater specificity than lipophilic dying, it suffers from deceased labeling efficiency [135]. Transmission electron microscopy (TEM) and cryoEM offer clear advantages, but processing and imaging are significantly more laborious. However, cryo-EM is currently the gold standard for the validation of EV structure and purity [136,137]. An emerging approach uses a combined correlative light and electron microscopy (CLEM) technique that allows for the nanometer resolution of EVs and fluorescent tagging of the protein of interest either in EVs or cells. A CLEM-based approach was used to track the intracellular fate of EVs and their cargo and successfully showed that EV cargo is released in the cytoplasm from late endosomes and lysosomes [138]. A summary of the different approaches discussed, and their advantages and limitations, is illustrated in Table 1. Similarly to other experimental approaches, more than one technique should be considered, and the best combination will depend largely on the hypothesis being tested and the end goal of the study. Finally, the immediate future of the EV field rests in the ability of newly developed technologies to analyze single EVs, analogously to single-cell approaches. One major advantage would be discovering surface markers that can identify populations of EV with similar functional identities. Single EV analysis would allow researchers to decipher the heterogeneity, find common trends and identify distinct differences in populations or sub populations of EVs. 

### 4.2. Approaches to Increase the Translatability of In Vitro Models to Study EV-EC Interactions

In order to better understand the capacity of EVs to be utilized as therapeutics or their potential use as disease biomarkers, more translatable approaches should be considered that better mimic in vivo conditions. The majority of approaches to study EVs include the addition of EVs to a static environment, such as a 2D monolayer of cells, and examining downstream effects through imaging techniques, gene or protein expression profiling and functional assays. These studies are essential first steps to characterizing EVs derived from a specific cell line and their impacts on a defined recipient cell type, as current in vivo approaches are challenging due to the multiple cellular sources of EVs that may lack specific markers, tracking difficulties and complex biodistributions. The importance of physiological conditions was demonstrated when HUVECS were cultured on a poly-dimethyl siloxane membrane to create elongated endothelial cells, and it showed that EVs expressed from elongated endothelial cells suppressed the activation of monocytes via the upregulation of anti-inflammatory miRNAs [143]. When considering 2D and 3D models, it was demonstrated that 3D models better mimic in-vivo EV characteristics of size distribution and cargo [144]. In fact, using modern 3D printing to create a scaffold that mimicked the basal membrane architecture for endothelial cells increased EV production when compared to a 2D cell culture [145].

For EV studies to become more translatable, more physiologically relevant conditions for EV–cell interactions should be considered. EVs secreted into the bloodstream likely have different uptake kinetics compared to zero flow conditions in culture media. Adopting systems that allow EC culture under various flow conditions is of key importance. One paper reports data collected when using a perfusion system to study EV interaction under flow conditions [146]. By using a modified microfluidic-based approach, one can combine the flow conditions with a system designed to capture cells and visualize, in real time, the secretion of EVs [147]. One group, using a liver on a chip and breast cancer derived EVs, was able to induce and confirm EndMT in liver sinusoidal endothelial cells [148]. Another group recently used a heart on a chip model to demonstrate the role of EVs derived from human microvascular endothelial cells in protection and rescue from ischemia-reperfusion injury [149]. The use of extracellular matrix (ECM) such as Matrigel has long been incorporated into research due to its importance in cell signaling to create more physiologically relevant in-vitro studies. Attention has been given to characterizing and determining what role EVs have in ECM function, and studies have demonstrated the importance of EVs in the maintenance and organization of the ECM [150]. Multiple groups have since made modified versions of different available ECM preparations with embedded EVs to determine interactions among cells, ECM and EVs. One group used electrospun nanofibrous scaffolds and immobilized EVs on the platform via the addition of ligands that tether the EVs to the scaffold. Using this approach, they demonstrated that EVs produced by placenta-derived mesenchymal stem cells increased angiogenic gene expression and survivability of ECs [151] Another group demonstrated that mesenchymal derived stem cells added to demineralized bone matrix prompted angiogenesis and bone regeneration in vivo by the subcutaneous addition of a EV modified scaffolds to 4 week old mice [152].

### 4.3. In Vivo Approaches to Studying EV–EC Interactions

Despite the advancements of in vitro modeling, in vivo approaches are the next required steps to gain better understanding of EV–cell interactions in an integrated system. Zebrafish have been used for their unique translucent property that makes live imaging less of a challenge. Zebrafish embryos were used to demonstrate EV uptake by circulating macrophages and to identify the cellular compartments in which EVs reside following their uptake [62]. Apart from zebrafish embryos, experimental approaches that measure EVs in vivo are rather limited to analysis of labeled EV biodistribution. Radiolabeling of EVs and imaging with single photon emission computed tomography (SPECT) have been used to determine the localization of endogenous EVs [153]. However, EV–cell interactions between subpopulations or individual EVs and cells are not possible using these approaches. A recent approach using CRISPR–Cas9 generated EC-EV lineage track mice by crossing CD63-emGFP with Cdh5-CreERT2 to generate endogenous CD63-tagged EVs from endothelial cells [154]. This approach presents an exciting opportunity to track, isolate and characterize endothelial derived EVs, and opens up opportunities for similar approaches using different EV markers.

## 5. Leveraging Extracellular Vesicles for Therapeutic Endothelial Targeting

### 5.1. Utilizing EVs as Biomarkers for Endothelial Damage with Prognostic Values

One of the primary cellular responses to damaged or compromised endothelium is EV release, making EC-EVs a critical early marker of vascular dysfunction [155]. Not surprisingly, a range of crucial pathologies affecting the vessel wall (obesity, hypertension, thrombosis and inflammation) display altered and often elevated numbers of EC-EVs [156]. EVs have the potential to serve as excellent biomarkers, since their count, content and origin might provide useful information about the pathophysiology of endothelial damage, however essential for a meaningful exploitation of circulating EVs as biomarkers is the differentiation of cellular origin [157]. EC-EVs are often distinguished using a common surface marker, CD31+ [52]. For example, endothelial dysfunction is an independent predictor of vascular disease; therefore, measurements of CD31+/annexin 5+ EVs are often assessed for diagnostic purposes [158]. It has been well documented that biomarkers of endothelial dysfunction include vascular cell adhesion molecule (VCAM-1), intercellular adhesion molecule (ICAM-1) and endothelial leukocyte adhesion molecule (ELAM-1) [159]. In addition, the presence of receptors, such as adenosine A2A receptor, on the membranes of circulating EVs in patients with coronary artery disease, may serve as biomarkers for endothelial dysfunction, although such EVs are likely released by cells other than endothelium [160,161]. However, what is truly needed to exploit EC-EVs for their diagnostic potential are tissue specific markers that will help in localizing the organ or tissue experiencing dysfunction. Recent studies have demonstrated that endothelium of the brain, heart and lungs can be differentiated based on their endothelial molecular signatures of pleiotrophin (Ptn), aquaporin (AQP7) and RAGE, respectively [17]. These recent advancements can improve our understanding and accuracy in pinpointing the sites of endothelial dysfunction. EVs have also been shown to be upregulated in patients with endothelial dysfunction [162], and steep increases in EV numbers are associated with pathological conditions of endothelial dysfunction such as arterial and venous thrombosis [157]. Currently, a few clinical trials are measuring EVs as biomarkers or direct effectors of thrombosis, tumor spread, endothelial dysfunction and inflammation [163]. As mentioned previously, contemporary research suggests that EC-EVs positive for TF have the potential to serve as the ideal biomarker for COVID-19 patients due to their prognostic value in correlating TF levels with the severity of the disease and mortality [116].

**Figure 3:**
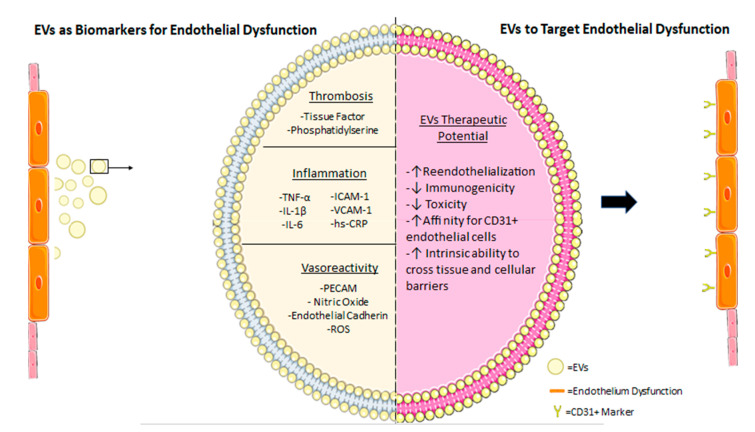
A graphical illustration depicting endothelial derived EVs theranostic potential as both biomarkers and agents of therapeutic delivery. EVs as biomarkers for endothelial dysfunction (**left**) summarizes known markers in thrombosis, inflammation and vasoreactivity that could be exploited for diagnostic purposes to attenuate endothelial compromise or damage. EVs to target endothelial dysfunction (**right**) summarizes the potential therapeutic benefit of utilizing endothelial derived EVs to target and promote dysfunctional endothelial regeneration.

One group perfectly illustrated the potential of EC-EVs as diagnostic tools by investigating multiple biomarkers of endothelium derived EVs, in patients with different degrees of disease severity [164]. The investigators measured biomarkers in 488 patients with coronary heart disease. Alongside the established biomarkers B-type natriuretic peptide and high-sensitivity C-reactive protein, they measured plasma levels of EC-EVs and found that an assessment of endothelial dysfunction by plasma level EC-EVs can independently predict future cardiovascular events in patients at high risk for coronary heart disease. Another study that evaluated EVs between patients diagnosed with cardiovascular disease compared to healthy controls concluded that the aforementioned biomarkers of endothelial dysfunction, as well as hs-CRP, IL-1, IL-6 and TNFα, were present at significantly higher levels in patients compared to healthy controls [165]. While there is much to do in the EC-EV biomarker discovery, existing data using total circulating EVs and the advances in discriminating EC-EVs from various vascular beds show great promise. In Figure 3, we summarize known biomarkers that are associated with pathologies of the endothelium (thrombosis, inflammation and vasoreactivity).

### 5.2. Using EVs to Target Endothelial Dysfunction

While several studies have highlighted EVs as contributors to the pathologies associated with endothelial dysfunction [157], recent evidence has suggested that EVs also have the potential to elicit a beneficial effect on injured endothelium [166] in acute and chronic vascular diseases [167]. These contrasting effects of EVs presumably stem from different subsets of EVs having different cargos, and thus indicate the potential to identify or develop populations of EVs which would target the injured endothelium and have potential therapeutic value. Figure 3 provides a summary of the potential therapeutic benefits of using EC-EVs to target endothelial dysfunction.

In contrast to their deleterious roles, EC-EVs can also exert beneficial effects, such as promoting EC survival. EC-EVs have inherent tissue repair-promoting properties that may be exploited therapeutically. For example, it has been established that endothelial cell derived EV populations contain a molecular cargo that has the ability to promote re-endothelialization of damaged vasculature [168] and in vivo studies have demonstrated that EC-EVs accelerated re-endothelialization in the early phase after endothelial damage in rat carotid artery [96]. The therapeutic potential of EC-EVs is a topic of intense research, both in the context of drug delivery and in regenerative medicine [169]. Two major therapeutic advantages of EC-EVs are their physiologic production and their low immunogenicity [170]. EVs also pose several advantages as drug delivery vehicles that make them outperform synthetic carriers [169]. Synthetic drug carriers, such as liposome nanoparticles, are often immunogenic and can exert toxicity even at very small concentrations, whereas therapeutic EVs are derived from either autologous or benign biological sources, and are therefore less likely to induce these adverse effects [171]. Additionally, EC-EVs have the intrinsic ability to cross tissue and cellular barriers [172]. Furthermore, some EVs may possess inherent targeting characteristics and display tropism for a particular cell or tissue [173]. For example, EC-EVs expressing the subunits β_5_ and β_4_ are preferentially targeted to Kupffer cells in the liver and CD31+ endothelial cells in the brain [174]. Although, as mentioned in the previous section, targetability can be further enhanced to desirable tissues of the brain, heart or lungs by ensuring EC-EVs have surface markers with affinities for Ptn, AQP7 and RAGE, respectively [17], thereby improving the efficaciousness of the intended therapeutic outcome. Functionalization of endothelial progenitor cell-derived EVs by click chemistry with a peptide, (RGDyk), which exhibits high affinity to integrin αvβ3 in reactive cerebral vascular endothelial cells after brain ischemia, enables more selective targeting of the injured EC only [175].

### 5.3. Challenges of EVs as Therapeutics

As mentioned beforehand, there is clear indication of a beneficial subset of EVs that can be isolated for therapeutic purposes. The translation of EVs into clinical therapies requires the categorization of EV-based therapeutics in compliance with existing regulatory frameworks [176]. Additionally, as the requirements for manufacturing, quality control and clinical investigations are further defined, it is of the utmost importance to establish whether EVs most appropriately function as active drug components or primarily serve as drug delivery vehicles [176]. To successfully translate from pre-clinical studies, methods of isolation and concentration must be further defined between studies such that there is no one-size-fits-all approach [177].

Strictly regarding endothelial dysfunction, despite promising research that has implicated the therapeutic potential of EVs as regulators of health in endothelial dysfunction, the use of EVs as drug delivery vehicles is still in need of further investigation to translate such experimental data into clinical therapeutics. It can be speculated that circulating EVs constitute an additional physiological mechanism to counter endothelium damage, one that may be altered in a diseased state [178]. The challenge is that there is a need to control the equilibrium between harmful and beneficial effects of EVs in the context of endothelial dysfunction [73]. This challenge encompasses the heterogeneity of EV populations, and the need for methods to enrich specific subset EVs of interest. A current issue is the absence of methods for the isolation, purification and preparation of EVs, which will vary depending on the research group and the cells from which the vesicles are derived [52]. The ways EVs are isolated and purified need to be standardized to validate reproducibility from one laboratory to another [179]. The development of new technical approaches and additional isolation techniques is required to increase the homogeneity of EV subpopulations. The specificity of ECs’ targeting mechanisms and the mechanisms for clearing ECs need to be better understood before EVs can be seriously considered as novel therapeutics for combatting endothelial dysfunction [180].

## 6. The Biological and Clinical Relevance of Endothelial EVs in COVID-19

COVID-19 infection caused by the novel severe acute respiratory syndrome coronavirus-2 (SARS-CoV-2) was shown to be associated with endothelial dysfunction manifested as increased formation of micro-thrombi, cytokine production by endothelium and deregulated immune responses [181]. Endothelial cells reportedly express both ACE2 and TMPRSS2 which makes them susceptible to direct viral entry and infection. However, both infected and uninfected endothelial cells may manifest dysfunction, which suggests that direct viral infection is not the solely responsible mechanism. EVs share some structural similarities with viruses, and recently it was found that viruses can exploit EVs for cellular exit or viral protein transfer, and EVs exploit viral entry mechanisms for cargo delivery [182,183]. Endothelium contributes significantly to the circulating EVs in blood and lymph, and it is conceivable to propose that endothelium-derived EVs could be responsible for the dissemination of pro-coagulant and pro-inflammatory molecules that can perpetuate the dysfunction initiated in infected cells. Indeed, proteomic analysis of the EV in plasma of patients with different degrees of COVID-19 severity showed distinct protein signatures that correlate with clinical manifestations of the disease [184]. In a separate study, Barberis et al. found that the proteome of the circulating EVs contained a large number of immune, pro-inflammatory and pro-coagulant proteins, and identified SARS-Cov-2 RNA in the exosomal cargo [185]. In a recent study, human lung endothelial cells exposed to the plasma of patients with severe disease and the plasma of patients with mild disease showed a significant difference in caspase3/7 and decreased cell survival in the former [186]. In the same study, authors showed that compared to asymptomatic controls, EVs isolated from patients on oxygen support with severe disease displayed increased protein expression for pro-thrombotic/endothelial injury factors such as t-PA, TF and vWF, along with proinflammatory proteins of the TNFα and IL 6 families. In another study, Rosell et al. showed that severe SARS-CoV-2 infection induces the release in circulation of EVs that harbor tissue factor and are likely to contribute to thrombosis in infected patients [116]. The presence of functional tissue factor in EVs showed correlation between its pro-coagulant activity and disease severity and was positively associated with mortality. Interestingly, in both studies, pro-thrombotic signatures were found in large circulating EVs and it was proposed, albeit without providing direct evidence, that they are primarily endothelium derived.

The data obtained so far strongly suggest the role of endothelium and endothelium-derived EVs in the pro-thrombotic and pro-inflammatory effects of SARS-CoV-2 infection in patients with COVID-19. Several studies showed that the protein profile of the circulating EV cargo is associated with disease severity and even mortality in COVID-19 patients. This makes EVs promising clinical biomarkers for disease prognosis and potentially for the response to future therapeutic approaches to mitigate the severe respiratory manifestation of the disease.

## 7. Concluding Remarks

In this review we revisited the extraordinary molecular and functional diversity of the vascular endothelium, which supports the notion that endothelial cells are programmed to serve specific functional adaptations of the vascular bed where they belong, which, in turn, is fine-tuned to the physiologic requirements of the adjacent stroma. We emphasized the physiological heterogeneity of endothelial cells along with their maladaptive plasticity in disease. Uniquely positioned at the interface between the fluid conduits and tissue parenchyma, endothelial cells control the bi-directional traffic of macromolecules, cells and EVs between the two compartments. Endothelium is therefore both a major contributor to circulating EVs and a gatekeeper of vesicular traffic between stroma and blood. Moreover, the EVs’ cargos and rates of biogenesis by the endothelium reflect the molecular and functional phenotypes of the endothelial cells of origin. In fact, EVs can disseminate molecules that originate in their parent cells to distant endothelial or non-endothelial cells and can alter their phenotypes. We focused on recent findings that support the roles of endothelial vesicles in health and disease, and highlighted the gaps in knowledge and the limitations of current experimental approaches and research paradigms. Finally, we discussed the potential of endothelial EVs as prognostic or diagnostic biomarkers and evaluated their promise as therapeutic targets or as tools for targeted delivery of other therapeutics. The recent advances in single cell biology made all of us re-evaluate the biological paradigms surrounding cellular identity and plasticity and much-needed approaches are emerging for studying the biology of single EVs. After all, personalized medicine for cardiovascular disease should not only be the prerogative for holistic treatment of individuals, but also for personalized healing of one endothelial cell and one EV at a time, which collectively should benefit the healing of the cardiovascular system.

## Figures and Tables

**Table 1:** Strengths and weaknesses of current imaging techniques used to visualize EV–EC interactions.

Imaging Approach	Advantages	Disadvantages
Widefield Fluorescent Microscopy [139]	Dyes are readily available and equipment is easy to use, less expensive than other methods	Resolution limits means direct visualization of individuals EVs is not possible
Confocal Microscopy [140]	Can Image live uptake and has higher resolution than conventional microscopy. Well-developed methods due to most common used approach to visualize EV–cell interaction	Resolution not high enough to see individual EVs on the smaller scale
Imaging Flow Cytometry [141]	High throughput capabilities allow for examination of millions of EV–cell interactions Analysis software allows for quantification of EV internalization and localization	Only allows the visualization of EV–cell interactions at specific moment in time Large file sizes due to capture of images
Electron Microscopy [142]	High enough resolution to visualize EV shape and structure	Must fix the sample before visualization
Super Resolution Microscopy [131]	Allows the visualization of individual EVs and can be used on live cells	Requires the use of specialized dye and greater optimization and equipment is not common in many labs

## Data Availability

Not applicable.
